# Life-history traits maintain the genomic integrity of sympatric species of the spruce budworm (*Choristoneura fumiferana*) group on an isolated forest island

**DOI:** 10.1002/ece3.11

**Published:** 2011-10

**Authors:** Lisa M Lumley, Felix AH Sperling

**Affiliations:** Department of Biological Sciences, CW 405 Biological Sciences Centre, University of AlbertaEdmonton, AB T6G 2E9, Canada

**Keywords:** Cypress Hills, *Choristoneura lambertiana*, *Choristoneura occidentalis*, hybridization, integrative taxonomy, phenology, pheromones, speciation, species delimitation

## Abstract

Identification of widespread species collected from islands can be challenging due to the potential for local ecological and phenotypic divergence in isolated populations. We sought to determine how many species of the spruce budworm (*Choristoneura fumiferana*) complex reside in Cypress Hills, an isolated remnant coniferous forest in western Canada. We integrated data on behavior, ecology, morphology, mitochondrial DNA, and simple sequence repeats, comparing Cypress Hills populations to those from other regions of North America to determine which species they resembled most. We identified *C. fumiferana*, *C. occidentalis*, *C. lambertiana*, and hybrid forms in Cypress Hills. Adult flight phenology and pheromone attraction were identified as key life-history traits involved in maintaining the genomic integrity of species. Our study highlights the importance of extensive sampling of both specimens and a variety of characters for understanding species boundaries in biodiversity research.

## Introduction

Islands have long fascinated biogeographers, ecologists, and evolutionary biologists, as they provide insights into the mechanisms that shape biological diversity (MacArthur and Wilson 1967; Brown and Lomolino 2000; Gillespie and Roderick 2002; Lomolino and Brown 2009). Island biogeography theory encompasses the biota of oceanic islands, isolated continental habitats, such as lakes or mountaintops (Hughes and Eastwood 2006; Thornton 2007), and habitats that have been fragmented through human activity (Harris 1984; Haila 2002; Thornton, 2007). However, this framework focuses on the number of species on an island as an equilibrium between colonization and extinction of species, implying little change in the species themselves, and is largely mute on the subject of species identification and delimitation on islands. Nonetheless, processes of divergence, hybridization, or speciation may occur on an island, particularly since adaptive traits may be under increased selection pressure to adapt to a new environment (e.g., Grant et al. 1996; Hughes and Eastwood, 2006). An increased propensity to hybridize and exchange genes may also be exhibited between closely related species in new contact on an island (e.g., Clarke et al. 1996; Grant et al. 1996). Our study focused on a continental island, Cypress Hills, where these challenges have complicated the identification of conifer-feeding species present in the spruce budworm (*Choristoneura fumiferana* Clemens 1865) complex (Lepidoptera: Tortricidae).

Cypress Hills is located on the border of southern Alberta and Saskatchewan in Canada, and is a forested continental island isolated from the nearest coniferous region by approximately 250 km (Chilton 2003). Coniferous tree species include pine (*Pinus contorta* Douglas ex Louden) and spruce (*Picea glauca*[Moench] Voss and *P. albertiana* S. Brown emend. Strong and Hills). *Picea albertiana* are hybrid forms of *P. glauca* (Moench) Voss and *P. engelmannii* Parry ex Engelmann (Strong and Hills 2006). Based on pollen records, Cypress Hills is a refugium that, due to its higher elevation and higher rainfall, has allowed *Picea* and *Pinus* species to survive, while surrounding regions developed into grassland somewhere between 12,000 and 14,000 years before present (Strong and Hills 2005).

The presence of these coniferous trees has allowed the colonization of insect species from the spruce budworm species group, a coniferophagous pest complex that ranges across the Nearctic region. This is an extremely well-studied species group of moths that includes *C. fumiferana*, which is the most destructive insect defoliator in North America (Volney and Fleming 2007) and has become a model organism for studying insect outbreak dynamics (e.g., Greenbank et al. 1980; Williams and Liebhold 2000; Royama et al. 2005; Régnière and Nealis 2007). Species within the complex are not all of equal economic importance, as some are less likely to enter an outbreak phase and cause widespread damage. The species also differ in their morphology, behavior, and bioregion association. However, species identification is a challenge since these differences are frequency related rather than discrete for each species (Harvey 1985; Dang 1992; Harvey 1997), and multiple characters are typically necessary for species identification. Although phenotypic traits work well for identification when spruce budworm species are found in their typical habitats, identifying species outside of their known range and in locations with nontypical environmental characteristics, such as Cypress Hills, can be very difficult. This is further complicated by the documented ability of spruce budworm species to hybridize and produce viable offspring (Harvey 1997). Genetic markers such as mitochondrial DNA (mtDNA) and simple sequence repeats (SSRs, also referred to as microsatellites) can help to identify some species, but the majority of the species can not be identified on the basis of these genetic markers alone as genetic clustering is less specific than the species boundaries. Therefore, putatively adaptive phenotypic traits such as life-history traits and morphology are still necessary to identify most of the species, requiring an integrative approach using multiple markers (Lumley and Sperling 2011).

The Cypress Hills habitat island is located between two major ecosystem regions hosting different species of the spruce budworm species complex (consult Lumley and Sperling 2011 for a species range map). About 400 km to the north and east is the boreal region with *C. fumiferana* and *C. pinus* Freeman 1953. To the west is a mountainous region with the remaining six species within the complex: *C. biennis*(Freeman 1967), *C. carnana* (Barnes and Busck 1920), *C. lambertiana* (Busck 1915), *C. occidentalis*(Freeman 1967), *C. orae*(Freeman 1967), and *C. retiniana* (Walsingham 1879). Described species that range geographically closest to Cypress Hills in the cordilleran region 250 km to the west include *C. occidentalis*, *C. biennis*, and *C. lambertiana*. A host plant connection between the boreal and cordilleran regions has been hypothesized by Strong and Hills (2005), potentially allowing refugial populations of several spruce budworm species to continue to exist for up to 14,000 years before present. These species are also known to migrate long distances, having been observed to disperse as far as 600 km (Dobesberger et al. 1983), which would allow more recent dispersal events from either the boreal or cordilleran regions to populate Cypress Hills.

The hybrid origin of one of the spruce species in Cypress Hills, *P. albertiana*, points toward the possibility that resident spruce budworm populations in Cypress Hills may also be hybrids, formed through secondary contact of “mainland” boreal and cordilleran species; all species within the spruce budworm complex have been shown to hybridize and produce fertile offspring in laboratory studies (Harvey 1997). With Cypress Hills containing only three conifer species there is also the possibility of increased rates of selection, leading to speciation, for species that have immigrated from other regions but do not originally prefer spruce (*P. albertiana* or *P. glauca*) or lodgepole pine. Colonizing or refugial species may also have to adapt to different environmental conditions in Cypress Hills compared to typical conditions in their normal range, again possibly leading to increased rates of selection and speciation.

With these considerations in mind, our objectives were to determine the identity of the spruce budworm species residing in Cypress Hills and to determine what characteristics may allow them to maintain their genomic integrity if there are multiple species. Putatively neutral markers (SSRs and mtDNA) were analyzed to measure gene flow and to determine whether there were any hybridization events among species or populations within Cypress Hills, as well as to assign individuals to previously delimited species by comparing them to “mainland” species. Adaptive traits (larval host plant, pheromone attraction, adult flight phenology, and adult forewing morphometrics) were surveyed to determine if there were any evolutionarily significant characters that may allow species to maintain their genomic integrity while existing in sympatry, as well as to assist in assigning Cypress Hills individuals to species by comparing their traits with those found in mainland species. Our work is intended to contribute to our understanding of spruce budworm species interactions as well as to explore appropriate methods for delimiting and identifying species on islands.

## Materials and Methods

### Collections for Cypress Hills

**Figure 1:**
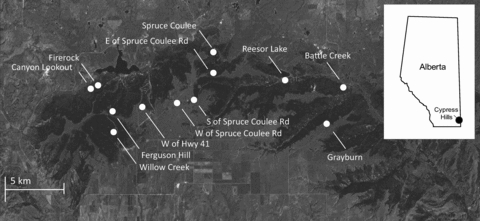
Cypress Hills collection locations from which specimens were sampled and analyzed. Photo of the Cypress Hills is from Google Maps (http://maps.google.com/).

As indicated in Fig. 1 and Table S1, collected specimens that were used in this study came from 12 locations in Cypress Hills, and included both larval and pheromone collections, which are further described below.

#### Larval collections

In 2005, larvae feeding on spruce were sampled from four locations in Cypress Hills (Table S1; Fig. 1), with two of the localities (Firerock and Battle Creek) having higher population numbers and providing more extensive collections. A few larval samples were also taken from spruce in 2008. Extensive searches were conducted across the region in 2006 and 2008 for larvae feeding on lodgepole pine, resulting in three individuals collected in 2008 from three localities. Larvae were fed original host foliage and reared to the adult stage. Larval head capsule coloration, larval diapause characteristics, and date of adult emergence were recorded along with typical locality information.

#### Adult collections

During July 8–11 in 2006, adults were collected using pheromone traps from across Cypress Hills in Alberta and Saskatchewan, of which seven locations were selected for further processing as listed in Table S1. Trap localities included locations containing mainly spruce or lodgepole pine as well as mixed stands. Two green unitraps (Contech, Victoria, BC) were set out 75 m apart and at a height of 2.75 m within each locality, with one trap containing *C. fumiferana* lure and one trap containing *C. pinus pinus* lure. The *C. fumiferana* pheromone lures consisted of 95:5 (*E*,*Z*)-11-tetradecenal (Contech). The *C. p. pinus* pheromone lures contained a 9:1 ratio of 85:15 (*E*,*Z*)-11-tetradecenyl acetates and 85:15 (*E*,*Z*)-11-tetradecen-1-ols (Silk et al. 1985) and were from the Canadian Forest Service. Vapona (Contech) was used as a killing agent. Blacklight traps were also placed out in these same locations for one trap night per locality over the three night collecting period, with ethyl acetate used as a killing agent. Longitude, latitude, elevation, and coniferous tree species were recorded for each location. Adults were transported back to the lab and frozen at –20°C to await further processing.

In 2008, pheromone traps were placed in nine localities across the region (Table S1; Fig. 1) with localities mainly being chosen on the basis of host plant stand. These included the same localities sampled for larvae in 2005 (Firerock, Spruce Coulee, Reesor Lake, Battle Creek), and many of the same localities sampled for adults in 2006 (Firerock, Willow Creek, east of Spruce Coulee Road, Spruce Coulee, Reesor Lake, Grayburn). Three localities mainly contained spruce, three mainly contained lodgepole pine and three localities were mixed stands, although all stands had some portion of either species. Three unitraps were set out for each locality, one trap containing *C. fumiferana* lure, one trap containing *C. p. pinus* lure, and one control trap containing no lure. The pheromone traps were placed in the field on June 27, 2008, and trap catches were collected and counted every 10 days until September 5, 2008, for a total of seven trap collections. Recorded location information included longitude, latitude, elevation, and coniferous tree species. Adults were placed at –20°C to await further processing. Voucher specimens and images were deposited at the University of Alberta in the E. H. Strickland Entomological Museum.

### Collections outside of Cypress Hills

Locality and collection information for spruce budworm species collected outside of Cypress Hills are compiled in Lumley and Sperling (2011). In addition, collection information for species that acted as outgroups for mtDNA analysis, *C. rosaceana* (Harris 1841), *C. conflictana* (Walker 1863), and *C. murinana* (Hübner [1796–1799]), is summarized in Lumley and Sperling (2011).

### Mitochondrial DNA

A 470-bp region of mtDNA was amplified and sequenced in both directions from the COI gene using the methods recorded in Lumley and Sperling (2010). This region was chosen as it allowed for the integration of data from previous studies (Sperling and Hickey 1994; Lumley and Sperling 2010, 2011). A total of 112 larval samples from 2005, 69 adult trap catch samples from 2006, and 296 larval and pheromone trap catch samples from 2008 were sequenced successfully for a total of 477 moths. From the 2006 collection, all specimens collected using the *C. pinus* lure were sequenced (*n*= 9), plus three locations were chosen from which 10 moths were sequenced from each of the *C. fumiferana* pheromone trap and blacklight trap catches. For the 2008 pheromone trap catches, at least three specimens were sequenced from every trap at every sampling date (including the control trap), unless fewer were collected in which case the one or two specimens available were sequenced.

mtDNA sequence was assembled and checked for ambiguities in Sequencher 4.0 (Gene Codes Corporation, Ann Arbor, MI), then aligned by eye in PAUP 4.0b10 (Swofford 2003). All haplotypes previously recorded from across North America from 1167 specimens (Lumley and Sperling 2011) were added to the analysis, including those sequenced for the same 470-bp region as well as 12 haplotypes sequenced for the full 2.3-kb region of COI and COII. Four outgroup specimens (2 ×*C. rosaceana*, *C. conflictana*, and *C. murinana*) were used. Sequence for the larger 2.3-kb region of mtDNA was included to maximize the phylogenetic informativeness and basal stability of the tree. Individual sequences from the Cypress Hills specimens were then reduced to unique haplotypes using MacClade v4.08 (Maddison and Maddison 2005). New haplotypes were deposited in Genbank (Table S2).

mtDNA sequence was analyzed using the same methods as in Lumley and Sperling (2011), using CIPRES portals v1.15 and v2.1 (Miller et al. 2009) for maximum parsimony, maximum likelihood, and Bayesian analysis. Maximum parsimony was analyzed in PAUP 4.0b10 (Swofford 2003) using a CIPRES wrapper around the PAUP heuristic search command, tree bisection and reconnection branch swapping, and 200 ratchet iterations. Strict and 50% majority rule consensus were calculated to generate a final tree. Maximum likelihood was analyzed in RAxML v7.0.4 (Stamatakis 2006), using the RAxML GTR + G + I model in CIPRES portal v1.15 and with 1000 rapid bootstrap inferences (Stamatakis et al. 2008). Bayesian analysis was conducted in MrBayes v3.2.1 (Ronquist and Huelsenbeck 2003), using the GTR + G + I model, the Markov chain Monte Carlo (MCMC) calculation running for 10,000,000 generations, trees sampled every 1000 generations, and the first 25% of trees being discarded as burnin. Convergence was confirmed by viewing the sump output. Trees were viewed in Treeview v1.6.6 (Page 1996).

### Microsatellite markers

Eight SSR loci were successfully amplified using the recommended conditions (Lumley et al. 2009), for 478 Cypress Hills specimens, including the same 477 specimens successfully sequenced for mtDNA. Amplified product was run using an ABI Prism 3730 DNA Analyzer, sized relative to Genescan LIZ-500 (Applied Biosystems, Foster City, CA), then checked and genotyped using GeneMapper 4.0 (Applied Biosystems). SSR genotypes from 1135 individuals that were analyzed in Lumley and Sperling (2011) were added to the data. These specimens were collected from across North America and included all species within the spruce budworm complex.

SSR data were analyzed in *structure* v2.3.2 (Pritchard et al. 2000) using the admixture model, with five broad geographic regions considered as sampling locality priors: (1) southern British Columbia, southwestern Alberta, and the western United States; (2) Rocky Mountains (north of Porcupine Hills, AB), northern British Columbia, Yukon, and Alaska; (3) coastal regions of British Columbia and Alaska; (4) east of the Rocky Mountains from Alberta to Newfoundland and the eastern United States; and (5) Cypress Hills. Inclusion of sampling location priors in the analyses can assist in the use of *structure* to make inferences on population structure if population membership and sampling location are correlated (Pritchard et al. 2010). Also, sample locations one to four were included in the analysis in Lumley and Sperling 2011, so it was appropriate to include them here so that comparisons could be made between the previous and current analyses. Allele frequencies were calculated using both the North American (Lumley and Sperling 2011) and Cypress Hills samples to determine if the Cypress Hills specimens were assigned to the same populations as the North American specimens and thereby were comparable. Allele frequencies were also calculated using the North American samples only, allowing population and individual assignments to be as similar as possible to those completed in Lumley and Sperling (2011), with Cypress Hills specimens assigned to populations based on the calculations for the North American samples. For both analyses, 10 iterations for each population size (*k*) equaling 1 through 13 were analyzed with MCMC running for 500,000 generations and initial burnin of 50,000 generations. For the first analysis, with Cypress Hills specimens included in allele frequency calculations, likelihood and Δ*K* were examined (Evanno et al. 2005) to determine the most likely number of populations. For the second analysis, with Cypress Hills specimens not included in allele frequency calculations, SSR assignment was visually compared to mtDNA and adaptive traits data for each individual, for *k*= 2 since this was the most likely number of populations calculated for the North American samples based on Δ*K*, and for *k*= 6 since this gave the most resolution for separating described species (Lumley and Sperling 2011).

### Morphology

For the pheromone trap collection completed in 2008, the forewings from all captured moths (*n*= 16,440) were examined and classified as brown, gray, or worn. Morphometric analysis was also performed on 398 specimens that had associated mtDNA and SSR data. These specimens were pinned, photographed, and imported into ImageJ 1.38x (Rasband 2006) to measure 25 morphometric forewing pattern elements as described by Lumley and Sperling (2010). Morphometric measurements were transformed by the log base 10 of *X*+ 1 and analyzed using linear discriminate analysis in Ginkgo v1.4 (De Cáceres et al. 2003). SSR population (*k*= 2) was the prior method for grouping individuals to determine if these populations could be distinguished using morphology.

### Combined data

Data gathered for Cypress Hills specimens were compared qualitatively to data gathered for each of the spruce budworm species collected throughout other regions of North America. This was to determine which species are most likely residing in Cypress Hills. All data types were also compared between specimens collected in Cypress Hills to determine if there were any genetic, morphological, or behavioral traits that differed between but corresponded within population types. This was to determine if there were any characters that may be contributing to the maintenance of the genomic integrity of species or populations. Cypress Hills specimens were also divided into four genetic types based on mtDNA and SSR (*k*= 2) data, these types being: (1) *Eastern*, for f- or p-lineage mtDNA + SSR population 1; (2) *Western*, for o-, oβ-, or bβ-lineage mtDNA + SSR population 2; (3) *Intermediate 1*, for o-, oβ-, or bβ-lineage mtDNA + SSR population 1; and (4) *Intermediate 2*, for f- or p-lineage mtDNA + SSR population 2. This was to evaluate whether there were any possible hybridization events between the two main populations, as determined by SSR assignment (*k*= 2). For specimens collected in 2008, these four genetic types were also plotted over time to determine if there was an intermediate flight period for hybrids. These genetic types were also mapped onto the linear discriminant analysis of the forewing pattern morphometrics to determine what population they were most similar to morphologically.

## Results

### Mitochondrial DNA

**Figure 2:**
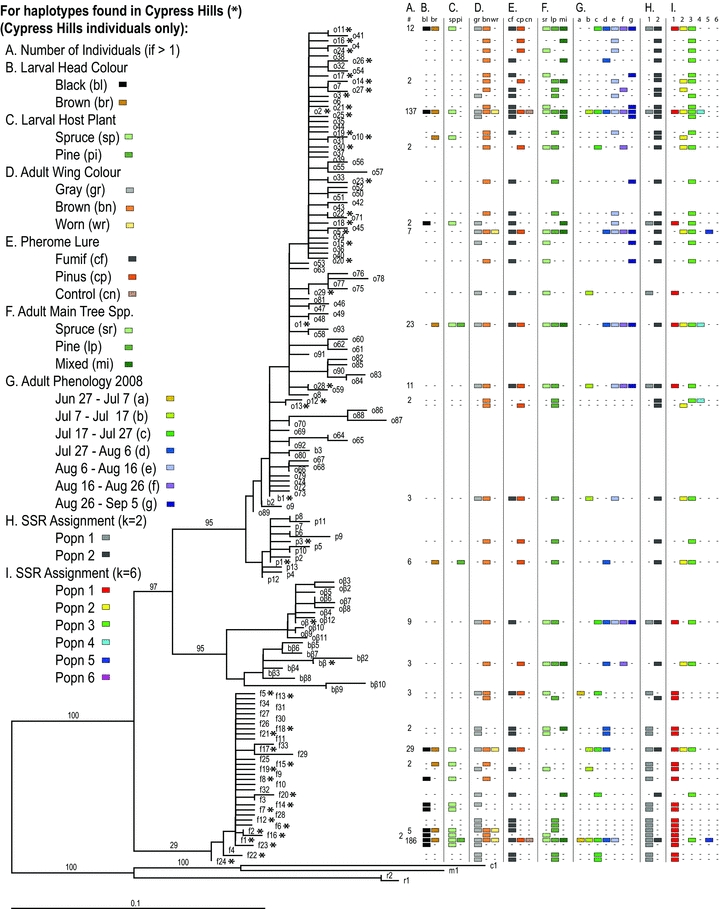
Maximum likelihood tree for 165 unique ingroup haplotypes found in the *Choristoneura fumiferana* species complex. The analysis included the full 2.3-kb region of cytochrome *c* oxidase I and cytochrome *c* oxidase II mitochondrial DNA (mtDNA) for 12 individuals and a 470-bp region of cytochrome *c* oxidase I mtDNA for 1632 individuals. Maximum likelihood bootstrap values and Bayesian support values are indicated for the main lineages. Haplotypes labeled by an asterisk (*) were found in Cypress Hills. Beside each haplotype found in Cypress Hills is phenotype information for specimens containing that haplotype (Cypress Hills specimens only), including: (A) number of individuals containing the haplotype; (B) larval head color; (C) larval host plant; (D) adult wing color; (E) pheromone attraction; (F) the main tree species in the locality where the specimens were collected; (G) adult phenology for 2008 pheromone trap collection; (H) SSR assignment for *k*= 2; and (I) SSR assignment for *k*= 6.

A total of 165 ingroup haplotypes (Table S2; Fig. 2) were included in the analysis, of which 142 were previously published (Sperling and Hickey 1994; Roe and Sperling 2007a; Lumley and Sperling 2010, 2011). These haplotypes represented 1630 individuals, and included all currently known spruce budworm species across their known range as well as the Cypress Hills specimens. From the 477 Cypress Hills specimens sequenced for the 470-bp region of COI mtDNA, a total of 49 ingroup mtDNA haplotypes were found, of which 23 were new and therefore unique to Cypress Hills (Table S2).

When restricted to the 470-bp region and with outgroups excluded, 71 characters were parsimony informative, 32 characters were variable but parsimony uninformative, and 367 characters remained constant. Maximum parsimony, maximum likelihood, and Bayesian analysis resulted in trees with similar topologies of major lineages and are in general agreement with those published in other studies (Sperling and Hickey 1994; Lumley and Sperling 2010, 2011). The maximum likelihood tree is shown in Figure 2. In comparing all locations that we have studied so far, Cypress Hills contains the highest amount of mtDNA diversity, with 49 mtDNA haplotypes and all five major mtDNA lineages (f, o, p, oβ, and bβ) as defined in Sperling and Hickey (1994). Two clusters are supported by bootstrap, one cluster exclusive to the f-lineage and the other cluster including the remaining named lineages (o, p, oβ, and bβ). The proportion of individuals associated with each lineage differs greatly, with the majority containing the f-lineage (*n*= 241) or o-lineage (*n*= 217) and smaller numbers of individuals containing the p-lineage (*n*= 7), oβ-lineage (*n*= 9), and bβ-lineage (*n*= 3). These lineages are associated with all known spruce budworm species, with the f-lineage primarily associated with *C. fumiferana*, with the p-lineage primarily associated with *C. pinus* in other regions, and with the o-, oβ-, and bβ-lineages associated with all western species (*C. occidentalis*, *C. biennis*, *C. orae*, *C. lambertiana*, *C. retiniana*, and *C. carnana*).

### Microsatellite markers

**Figure 3:**
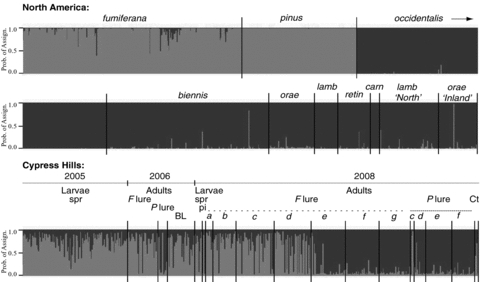
Probability of microsatellite assignment using *structure* analysis (*k*= 2), with North American samples (excluding all but 10 Cypress Hills specimens, as per Lumley and Sperling, 2011) grouped by species, and Cypress Hills samples grouped by collection information (collection year, collection taken as larvae or adults, larval host plant, adult trap type, and collection date for 2008 phenology study). *lamb*=*lambertiana*; *retin*=*retiniana*; *carn*=*carnana*; spr = spruce; pi = lodgepole pine; *F* lure =*C. fumiferana* lure; *P* lure =*C. pinus* lure; BL = blacklight; Ct = Control; *a*–*g*= collection dates (*a*= June 27– 7; *b*= July 7–17; *c*= July 17–27; *d*= July 27–August 6; *e*= August 6–16; *f*= August 16–26; *g*= August 26–September 5).

When Cypress Hills specimens were included in the calculation of allele frequencies using *structure* (Pritchard et al. 2000), the likelihood values and Δ*K* (Evanno et al. 2005) indicated that the most likely number of populations is two (Figs. 2 and 3). This result was the same as for the North American samples only (Lumley and Sperling 2011), indicating that populations in Cypress Hills could be compared to those in surrounding areas. When Cypress Hills specimens were not included in the calculation of allele frequencies, *k*= 2 indicated that the two populations were both in Cypress Hills (Fig. 3). Based on the North American samples, Population 1 encompasses species residing in the boreal regions and the eastern United States (*C. fumiferana*, *C. pinus*) and Population 2 encompasses the remaining species residing in the west (*C. occidentalis*, *C. biennis*, *C. orae*, *C. lambertiana*, *C. retiniana*, and *C. carnana*) (Fig. 3).

We also examined the assignment of Cypress Hills specimens at *k*= 6 (Fig. S1), as this was the number of populations at which the most North American species were delimited using the eight SSR markers (Lumley and Sperling 2011). In the North American study (Lumley and Sperling 2011), individuals were identified to species on the basis of phenotypic traits (life history, behavior, ecogeography, and morphology), and the SSR analysis grouped these same individuals as Population 1 (*C. fumiferana*), Population 2 (Western A), Population 3 (Western B), Population 4 (*C. lambertiana*), Population 5 (*C. pinus*), and Population 6 (*C. retiniana*). It was determined that Western A and Western B contained the western species, mainly *C. occidentalis*, *C. biennis*, *C. orae*, *C. carnana*, as well as several specimens identified as *C. lambertiana* and *C. retiniana*. Using the same individuals analyzed for the North America study (Lumley and Sperling 2011) to assign the Cypress Hills individuals, it was found that Cypress Hills individuals were assigned to Population 1 (*C. fumiferana*, *n*= 269), Population 2 (Western A, *n*= 28), Population 3 (Western B, *n*= 175), Population 4 (*C. lambertiana*, *n*= 4) and Population 5 (*C. pinus*, *n*= 2). No individuals were found that were assigned to Population 6 (*C. retiniana*).

### Life history and morphology

Life-history, behavioral, and morphological data collected for the Cypress Hills specimens were compared qualitatively to data gathered for North American species. Life-history and behavioral data included larval host plant, larval diapause, pheromone attraction, and adult flight phenology. Morphological data included larval head capsule and adult forewing coloration.

The larval host plant for larvae collected in Cypress Hills was almost exclusively spruce (Fig. 2). Several spruce budworm species are associated with spruce, including *C. fumiferana* (white spruce), *C. biennis* (Engelmann spruce), and *C. orae* (Sitka spruce) (Harvey 1985). *Choristoneura occidentalis* has also been found to occasionally feed on spruce (Harvey 1985). Very few larvae (*n*= 3) were found on lodgepole pine despite extensive searching in Cypress Hills. In other regions, *C. lambertiana* is the main species associated with lodgepole pine, although *C. pinus* has also been found to occasionally feed on this host plant (Harvey 1985). Most Cypress Hills larvae were collected early enough (second to fourth instar) to determine that they went through only 1 year of larval diapause. *Choristoneura biennis* larvae go through a second larval diapause, a fixed trait for this species (Nealis 2005). *Choristoneura orae* larvae may also go through a second larval diapause (Harvey 1967), and the remaining species typically undergo only 1 year of larval diapause (Harvey 1985).

Adult *Choristoneura* males were collected in Cypress Hills using both the *C. fumiferana* and *C. pinus* pheromone lures in 2006 and 2008 (Fig. 2). Counts were only made for the 2008 collection, with a total of 16,210 moths caught using the *C. fumiferana* lure and 224 moths caught with the *C. pinus* lure. Species typically attracted to the *C. fumiferana* lure are *C. fumiferana*, *C. biennis*, *C. occidentalis*, and *C. carnana* (Lumley and Sperling 2011). Species typically attracted to the *C. pinus* lure are *C. pinus*, *C. retiniana*, *C. lambertiana* (Lumley and Sperling 2011), and most likely *C. orae* (Harvey 1985, unpubl. data). Phenology data from 2008 indicate that adults fly from late June to mid September. This covers the flight period of all species within the complex (Freeman 1967; Powell and De Benedictis, 1995).

For color pattern, larvae had either dark brown to black (*n*= 184) or lighter brown (*n*= 33) head capsules (Fig. 2). In other regions, *C. fumiferana* larvae generally have dark brown to black head capsules, and the remaining species typically have head capsules that are either lighter brown or lighter brown with darker lateral stripes (Harvey and Stehr 1967; Lumley and Sperling 2010). Cypress Hills adults had either gray or brown forewings. Species mainly associated with gray forewings are *C. fumiferana*, *C. biennis*, and male *C. orae* (Freeman 1967). *Choristoneura fumiferana* females and *C. biennis* males and females may also have brown wings (Freeman 1967). *C. occidentalis*, *C.carnana*, *C. pinus*, *C. lambertiana*, and female *C. orae* typically have brown forewings (Freeman 1967). There were no specimens with tawny wings resembling *C. retiniana* (Freeman 1967).

### Morphometrics

**Figure 4:**
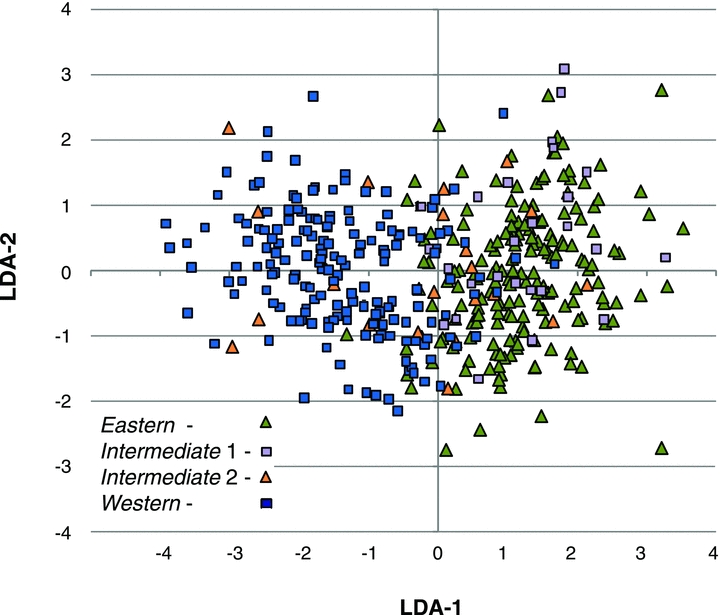
Linear discriminant analysis of 47 morphometric characters for Cypress Hills specimens, grouped a priori by SSR population (*k*= 2). Specimen coordinates are labeled to indicate the four genetic combinations: *Eastern*= f- or p-lineage mtDNA + SSR population 1; *Western*= o-, oβ- or bβ-lineage mtDNA + SSR population 2; *Intermediate 1*= o-, oβ- or bβ-lineage mtDNA + SSR population 1; and *Intermediate 2*= f- or p-lineage mtDNA + SSR population 2.

Forty-seven morphometric characters were measured for 398 specimens, with SSR population (*k*= 2) as the prior method for grouping individuals, to determine if these two populations could be identified using forewing color and pattern alone. This also identified which population the intermediate specimens were most similar to in forewing characters. The first canonical discrimination function explained 100% of the variation between the two populations (Fig. 4), and Wilk's Lambda test of functions was significant (*P* < 0.05).

Under no selection, training set resubstitution evaluation and leave-one-out evaluation assigned 352 (88%) and 335 (84%) individuals to the correct population, respectively. Under stepwise selection, the number of characters analyzed was reduced to 11 of the 47 that were measured, and resulted in training set resubstitution evaluation and leave-one-out evaluation correctly identifying 347 (87%) and 342 (86%) individuals to the correct population, respectively. Therefore, the number of characters measured can be decreased without reducing correct identification. As indicated on the graph (Fig. 4), the two SSR populations form clusters but there is some overlap between them.

Figure 4 shows individuals mapped using four different symbols, based on their genetic combination of mtDNA and SSRs (*Eastern*, *Western*, *Intermediate 1*, *Intermediate 2*). This reduced the number of individuals overlapping between the “pure” populations (*Eastern* and *Western*), though some overlap is still present. It also showed that *Intermediate 1* specimens were clustered with population 1, whereas *Intermediate 2* specimens were more scattered throughout both the population 1 and population 2 clusters.

### Combined data

**Figure 5:**
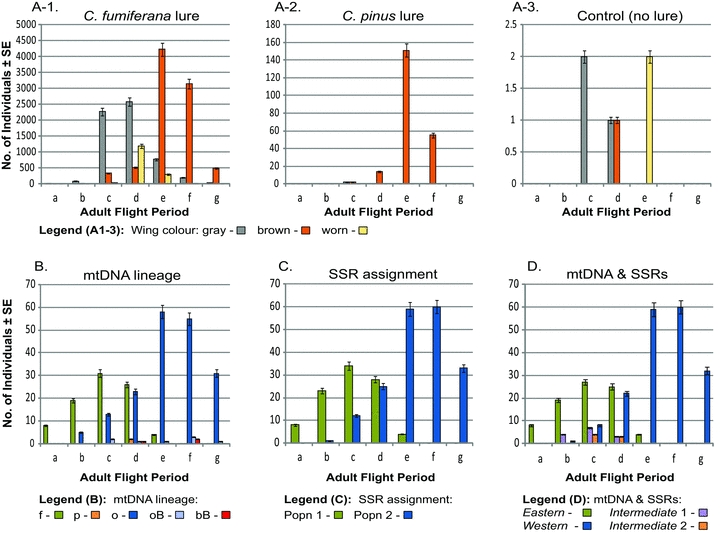
Phenology histograms for the 2008 adult samples, showing correlation with: (A1–3) wing coloration, separated by adult pheromone lure type; (B) mtDNA lineage; (C) SSR assignment (*k*= 2); (D) mtDNA and SSR assignment combined, giving four genetic combinations: *Eastern*= f- or p-lineage mtDNA + SSR population 1; *Western*= o-, oβ- or bβ-lineage mtDNA + SSR population 2; *Intermediate 1*= o-, oβ- or bβ-lineage mtDNA + SSR population 1; and *Intermediate 2*= f- or p-lineage mtDNA + SSR population 2. Letters *a*–*g*= collection dates (*a*= June 27– 7; *b*= July 7–17; *c*= July 17–27; *d*= July 27–August 6; *e*= August 6–16; *f*= August 16–26; *g*= August 26–September 5).

mtDNA, SSR assignment, life history, and morphological traits were compared qualitatively as combinations within and between individuals from the Cypress Hills. For the 2008 collections, there were two spruce budworm populations in Cypress Hills that were both attracted to the *C. fumiferana* lure but differed in adult phenology. An early-flying group, collected primarily from June 27 to August 6, were mainly gray-winged moths with f-lineage mtDNA and population 1 SSR assignments (Figs. 3 and 5). The early-flying group most resembles *C. fumiferana* based on this combination of data. A late-flying group, collected primarily from August 6 to September 5, was mainly brown-winged moths with o-lineage mtDNA and population 2 SSR assignments (Figs. 3 and 5). The late-flying group most resembles *C. occidentalis* based on this data combination. Our data also indicate that there is a third population attracted to *C. pinus* lure. This mid-flying group, collected primarily from July 27 to August 26, is very similar in forewing color and pattern to the late-flying group, containing mainly brown-winged moths with o-lineage mtDNA and population 2 SSR assignments (Figs. 3 and 5). The mid-flying group most resembles *C. lambertiana* based on this combination of data.

Comparison of the 2005 larval collections, 2006 adult collections from July 8 to 11, and 2008 adult collections indicated that the 2005 and 2006 collections were most similar to the 2008 early-flying adults (*C. fumiferana*) (Fig. 3). However, there were still a variety of mtDNA haplotypes and assignments to both SSR populations (*k*= 2) within the 2005 and 2006 collections. The 2008 control pheromone traps, containing no pheromone lure, caught a total of six moths: three moths were most likely *C. fumiferana*, being early-flying, having either gray wings or worn wings during the middle of the flight season, f-lineage mtDNA, and population 1 SSR assignments (*k*= 2); two moths were most likely *C. occidentalis,* being late-flying, with one being brown and the other gray, and both having o-lineage mtDNA and population 2 SSR assignments; and one gray moth was an intermediate, having f-lineage mtDNA but population 2 SSR assignment.

There were rare specimens collected with p-, bβ-, or oβ-lineage mtDNA for which it was difficult to determine whether they were part of the three main species or represented separate groups. Specimens with p-lineage mtDNA were collected with *C. pinus* lure within the same time frame as *C. lambertiana*, and they had similar adult forewing features, population 2 SSR assignment at *k*= 2, and population 2 or 3 SSR assignment at *k*= 6. This differs from the North American samples since specimens with p-lineage mtDNA from east or north of Cypress Hills are highly associated with SSR population 1 at *k*= 2 (Eastern) and SSR population 5 at *k*= 6 (*C. pinus*) (Lumley and Sperling 2011). Specimens with bβ-lineage mtDNA were also collected with the *C. pinus* lure, had a mid-summer flight (July 27–August 26), were brown winged, and were assigned to SSR population 2 at *k*= 2 and SSR population 2 or 3 at *k*= 6. Individuals with oβ-lineage mtDNA were collected with the *C. fumiferana* lure, but were mainly late flying (July 17–September 5) with either brown or gray wings, were assigned to SSR population 1 or 2 (mainly population 2) at *k*= 2, and were assigned to SSR population 1 or 3 (mainly population 3) at *k*= 6.

For the 2008 phenology data, individuals were classified under four genetic types to determine the possibility of hybridization events in Cypress Hills (Fig. 5D). The majority of specimens were either *Eastern* (*n*= 83) or *Western* (*n*= 182), with the *Eastern* specimens defined by having f- or p-lineage mtDNA and SSR assignment to population 1 (*k*= 2), and the *Western* specimens defined by having o-, oβ-, or bβ-lineage mtDNA and SSR assignment to population 2 (*k*= 2). There were also fourteen specimens with the *Intermediate 1* genetic type, defined by having o-, oβ-, or bβ-lineage mtDNA and SSR assignment to population 1 (*k*= 2), and seven specimens with the *Intermediate 2* genetic type, defined by having f- or p-lineage mtDNA and SSR assignment to population 2 (*k*= 2). Both *Intermediate 1* and *Intermediate 2*, which may refer to two hybrid genetic types, were mid-summer fliers in that they were collected as adults from July 7 to August 6. During this time period, both the *Eastern* and *Western* populations were flying.

## Discussion

Many factors must be taken into account when delimiting and identifying species on islands, including the propensity for species to undergo adaptive radiations and hybridization events in new, geographically constrained ecotypes (e.g., Grant et al. 1996; Schluter 2000; Seehausen 2004; Petren et al. 2005). This challenge was faced in identifying spruce budworm individuals to species in Cypress Hills, with the added difficulty that “mainland” life-history traits or ecogeographical features are typically necessary for species identification. Although isolated, Cypress Hills is situated between two major ecogeographical regions, the boreal and cordilleran, each of which contains different spruce budworm species. Considering the biogeographical history of the region (Strong and Hills 2005), along with the ability of spruce budworm to migrate long distances (Dobesberger et al. 1983), it is plausible that any of the species in the group occur in Cypress Hills. The documented ability of spruce budworm species to hybridize (Harvey 1997), adds the possibility of further complexity in the Cypress Hills through secondary contact between previously allopatric species.

By integrating life history, behavior, morphology, and genetics we determined that there are at least three spruce budworm species in Cypress Hills, each with a distinct flight period. The early-flying group is *C. fumiferana*, with gray forewings, f-lineage mtDNA, and assignment to SSR population 1 (*k*= 6). The late-flying group is most likely *C. occidentalis*, with brown forewings, o-lineage mtDNA, and assignment to SSR population 2 or 3 (*k*= 6). Identification of the late-flying group was partly through a process of elimination. No larvae were found in Cypress Hills that went through second diapause and this is a fixed character for *C. biennis* (Nealis 2005). *Choristoneura carnana*, the remaining species attracted to the *C. fumiferana* lure, has only been found in California, southern Oregon, and Arizona, which are geographically distant from Cypress Hills, and specimens collected in Cypress Hills had adult forewing features that were more similar to *C. occidentalis* than to *C. carnana*. Cypress Hills also contains a smaller mid-summer flying third group that is attracted to the *C. pinus* lure and most resembles *C. lambertiana.* More specifically, it resembles *C. lambertiana*“North”, a group that was identified in Lumley and Sperling 2011. All individuals within this group had brown forewings as adults, and most had o-lineage mtDNA and assignment to SSR population 2 or 3.

There were also some individuals that had p- or bβ-lineage mtDNA. Of particular interest, there is evidence for the decoupling of p-lineage mtDNA from the usual nuclear genome of *C. pinus*, since SSR population assignment for Cypress Hills specimens containing the p-lineage did not correspond to that of individuals collected in other regions of North America. It is possible that there were previously *C. pinus* individuals residing in Cypress Hills that eventually hybridized with *C. lambertiana* to produce this unusual genotype.

Considering the propensity for species to hybridize in new, geographically confined regions (Seehausen 2004), as would be the case for Cypress Hills, it is surprising that the two main species, *C. fumiferana* and *C. occidentalis*, have remained separate with relatively few hybrids, as determined by the proportions of the four mtDNA and SSR combinations (*Eastern*, *Western*, *Intermediate 1*, *Intermediate 2*). Several traits may allow these species to maintain their genomic integrity. Although these two species are both attracted to the *C. fumiferana* lure, it is possible that missing components in the artificial lure would normally allow individual discrimination. The species are morphologically different, as determined by simple wing-color scoring as well as morphometric analysis. Most importantly, they are phenologically different. Although they have an overlapping flight period, the number of individuals flying from each species differs substantially, with rapid transition. This may reduce the opportunity for them to hybridize. Interestingly, all identified hybrids had an intermediate flight period that was within the period of time during which both *C. fumiferana* and *C. occidentalis* were flying. Laboratory experiments on spruce budworm hybrids have also found hybrids to undergo intermediate development between that of their parents, which may result in intermediate flight periods (Smith 1953; Harvey 1967; Volney and Liebhold 1985).

The larvae collected in 2005 are more genetically similar to *C. fumiferana* than to *C. occidentalis*. There are three possibilities that may explain the lack of *C. occidentalis* larval samples. First, the *C. occidentalis* individuals collected as adults in 2008 may have migrated from elsewhere, most likely from southern or western regions (e.g., Montana, Idaho, Washington, British Columbia, southern Alberta) where we collected *C. occidentalis* in high numbers and observed severe host defoliation within these same years. Cypress Hills is within the flight range of some of these regions. Second, within Cypress Hills, *C. occidentalis* may have been feeding in localities or on host plants that were not sampled as larvae. All larvae were sampled from lower, accessible branches of the host plant, so if *C. occidentalis* larvae were feeding in other regions of the host, or residing in areas of Cypress Hills that were not sampled, then they may not have been collected in proportions that represented their numbers as adults. Third, the 2005 larvae were sampled early in the season when they were second to third instar so that diapause characteristics could be studied. Late-flying *C. occidentalis* individuals may have still been in diapause or in the process of migrating to the bud to feed, and therefore may have been missed during collection. This is of particular interest as, if *C. occidentalis* is residing in Cypress Hills, then it has adapted to feed on a nontypical host (white spruce or hybrid spruce) that is possibly nutritionally and phenologically different from the typical host (Douglas-fir).

Overall, our results demonstrate the importance of collecting samples at intervals throughout the overall flight period for studies focused on identifying and monitoring species or populations. This principle applies to biodiversity studies, insect pest and invasive species monitoring, and almost any study focused on systematics or population genetics. Specimens collected in 2005 and 2006, along with prior collections by Sperling and Hickey (1994), indicated that there were different co-occuring mtDNA lineages, but the majority of these specimens resembled *C. fumiferana*. A shortage of specimens and few biological differences made it unreasonable to separate out additional species. By sampling the full flight period over 10-day intervals in 2008, we were able to determine that there were additional species with different biological characteristics residing in Cypress Hills that were missed, or misrepresented in proportion, by collecting early, single-period samples (early June for larvae, early July for adults).

This study highlights the importance of using integrative methods and broad sampling for species or population delimitation, a contentious issue amongst taxonomists (e.g., Dayrat 2005; Will et al. 2005) that is nonetheless increasingly being supported through case-focused research (e.g., Roe and Sperling 2007b; Ross et al. 2009; Schlick–Steiner et al. 2010). Without combining behavioral, morphological, and genetic traits, further delimitation of species within the Cypress Hills would have remained ambiguous, as they were with the use of mtDNA alone (Sperling and Hickey 1994). This is particularly true for the identification of closely related, sympatric species on islands where typical ecogeographical and life-history traits are unavailable.

## Supporting Information

**Figure S1.** Probability of microsatellite assignment using structure analysis (*k* = 6), with North American samples (excluding all but 10 Cypress Hills specimens, as per Lumley and Sperling 2011) grouped by species, and Cypress Hills samples grouped by collection information (collection year, collection taken as larvae or adults, larval host plant, adult trap type, and collection date for 2008 phenology study).

**Table S1.** General location information for analyzed samples collected in Cypress Hills, Alberta, Canada.

**Table S2.** mtDNA haplotypes with corresponding GenBank accession numbers and references.
